# The effect of whole-body vibration training on lean mass: A PRISMA-compliant meta-analysis

**DOI:** 10.1097/MD.0000000000008390

**Published:** 2017-11-10

**Authors:** Hengting Chen, Jianxiong Ma, Bin Lu, Xin-long Ma

**Affiliations:** a Tianjin Hospital; b Tianjin Medical University; c Biomechanics Labs of Orthopaedics Institute, Tianjin Hospital, Tianjin, People's Republic of China.

**Keywords:** lean mass, meta-analysis, muscle mass, WBV

## Abstract

**Background:**

Whole-body vibration training (WBVT) confers a continuous vibration stimuli to the body. Although some reports have discussed the effects of whole-body vibration (WBV) on bone mineral density and muscle strength, study of WBV effects on lean mass have not been determined. The purpose of the present meta-analysis was to evaluate published, randomized controlled trials (RCTs) that investigated the effects of WBVT on lean mass.

**Methods:**

We identified only RCTs by searching databases, including Web of Science, PubMed, Scopus, Embase, and the Cochrane Library from inception to March 2017. Data extraction, quality assessment, and meta-analysis were performed.

**Results:**

Ten RCTs with 5 RCTs concentrating on older people, 3 on young adults, and 2 on children and adolescents were included. We additionally explored the effect of WBVT on postmenopausal women (6 trials from the 10 trials). Significant improvements in lean mass with WBVT were merely found in young adults (*P* = .02) but not in other populations compared to control group.

**Conclusion:**

The effect of WBVT found in the present meta-analysis may be used in counteracting the loss of muscle mass in younger adults. Moreover, optimal WBVT protocols for greater muscle hypertrophy are expected to be investigated.

## 1 Introduction

As average life expectancy has shown to prolong over the years in both developed and less developed countries, there is also a concurrent increase in health-related problems.^[1]^ Sarcopenia is a term derived from Greek words sarx (flesh) and penia (loss), which was firstly used by Rosenberg in 1989 to describe the decline in muscle mass among older people.^[2]^ The total decline in the muscle mass between age 40 and 80 years ranges from 30% to 50% and the annual decline in functional capacity is reported to be 1% to 2% after the age of 50 increasing to as much as 3% after the age of 60.^[3,4]^ To our knowledge, there are a crowd of reasons could result in muscle mass loss, such as physical inactivity, reduced protein intake, and many acute or chronic diseases, which could not merely happen in older people but in younger population. Muscle mass loss/sarcopenia is highly associated with osteopenia/osteoporosis, fracture, and many other diseases, and it will increase the risk for disability, hospitalization, and death in older adults.^[5–7]^ The ultimate remedy for sarcopenia yet has to be found, some interventions have proven their merits and might be of practical use in clinical practice, among which different types of physical exercise (eg, resistance training, high-impact aerobic activity, and Ving Tsun Chinese martial art) have been used to alleviate age-related muscular deterioration.^[8,9]^ However, the traditional impact exercise approach is strenuous and may increase the risk of fall, particularly when the exercise is unsupervised.^[10,11]^ Furthermore, some patients may have illnesses that prevent them from participating in vigorous impact exercise training.^[12,13]^ Therefore, there is an urgent need to develop safe and effective alternative methods to maintain muscle mass and to prevent sarcopenia.

Whole-body vibration (WBV) is a new way of exercise which uses mechanical stimuli generated by a vibrating platform and transmitted through the body where they load the body and stimulate sensory receptors.^[14]^ Previous studies have showed the obvious effect of WBV on improving muscle strength in mice and older adults.^[15,16]^ Muscle strength and lean mass are highly interrelated.^[17]^ Therefore, whole-body vibration training (WBVT) is expected to play a positive role on muscle mass. So far, numerous intervention studies have used WBVT to increase muscle mass of human body.^[18]^ However, some of them have shown controversial results. Moreover, the most effective vibration training protocols for clinical use in the treatment of sarcopenia are yet to be identified, which especially refer to amplitude, frequency, acceleration, and cumulative volume. Therefore, to more objectively advance our knowledge of its role in clinical practice, we conducted a meta-analysis of WBV effect on lean mass or muscle mass in humans.

## 2 Methods

### 2.1 Systematic literature search

**Figure 1:**
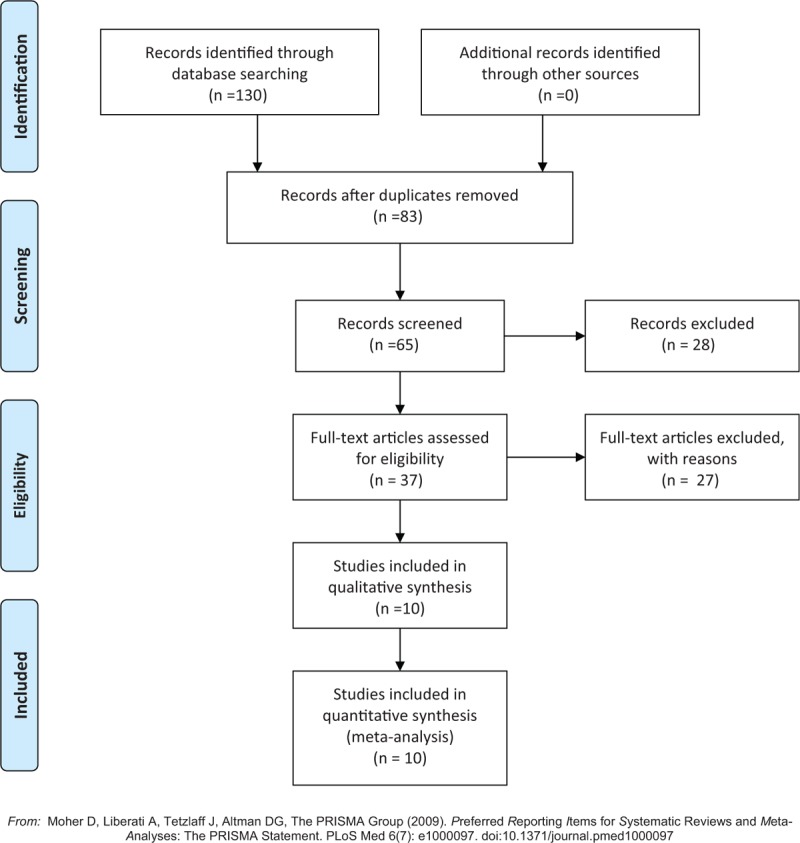
Flow chart of the selection of studies.

The following electronic databases were searched from inception to March 2017: Web of Science, PubMed, Embase, Scopus, and the Cochrane Library. The following keyword combinations were used “whole body vibration” OR “WBV” AND “muscle mass” OR “lean mass.” Figure 1 shows a flow diagram of the results from the entire search process.

### 2.2 Inclusion and exclusion criteria

Since this study was a meta-analysis of published studies, ethical approval was not required. Inclusion criteria were: randomized controlled trials (RCTs); outcomes relating to lean mass or muscle mass; subjects were human who were not restricted based on age, sex, race, or physical activity levels; vibration was applied to the whole body via a platform with no restriction on training position, the frequency (hertz), amplitude (millimeters), magnitude (vibration acceleration due to gravity, g), study duration and sessions of WBV; acceptable control interventions types including no treatment, sham vibration (audible sound with no mechanical vibration), and exercise interventions; and articles were published and in full length.

Exclusionary criteria were: non-English language papers; papers where only animal data were included; literature or systematic reviews; and non-RCTs.

### 2.3 Data extraction and risk of bias assessment

**Figure 2:**
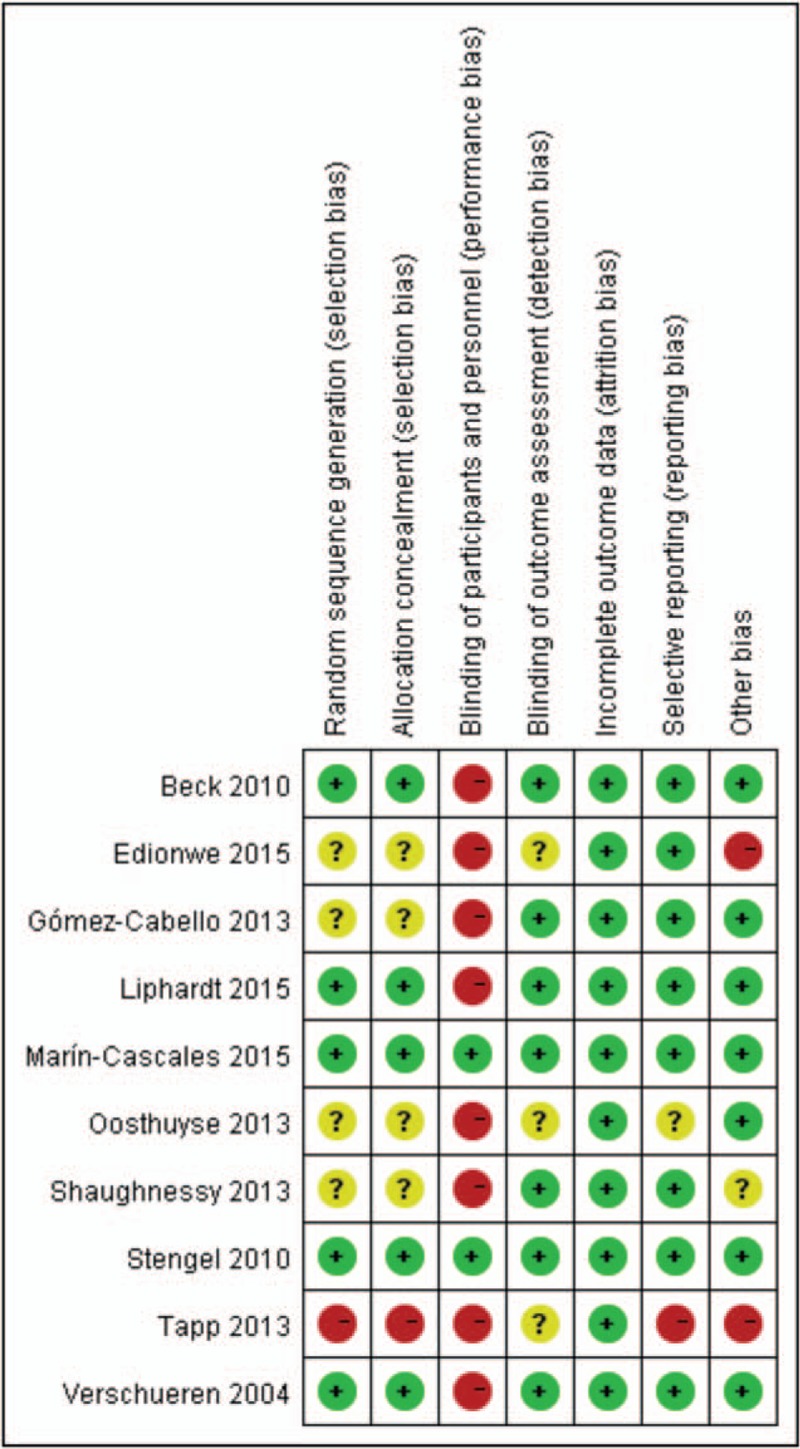
The summary of bias risk of randomized controlled trials.

A standard data extraction form was designed to extract the relevant data from the included articles. Two reviewers used this form to collect the information from studies independently. The extracted data included any relevant information regarding the trials’ characteristics, lean mass or muscle mass outcomes, and methodological quality. Data were extracted separately for 65 years of age or older, younger adults (between 18 and 65 years old), children, and adolescents, so that separate analyses can be performed for each population. Moreover, postmenopausal women were solely extracted from the included studies. Pooling these populations would introduce unwanted clinical heterogeneity, because physiologically different muscle metabolic processes occur in these populations. In children and adolescents, muscle is being developing, and their muscle mass typically increases over time and in response to effective therapies. In other groups, muscle mass generally plateaus and would be expected to increase in response to effective interventions. Seven aspects of the studies related to the risk of bias were assessed, following the instructions in the Cochrane Handbook for Systematic Reviews of Interventions (Fig. 2).

### 2.4 Statistical methods

Review Manager Software for Windows (Version 5.3. Copenhagen: The Nordic Cochrane Center, The Cochrane Collaboration, 2014) was used to analyze study results. A random-effects model was used for all analyses, as clinical heterogeneity was assumed to exist because of differences in study methods. The effect size (ES) of the intervention was the difference in muscle mass or lean mass after the WBVT and in control participants. On account of that the protocols for WBVT are fairly various among the respective studies, the inverse variance method was used to standardize the mean differences by dividing the values with their corresponding SD. The standardized mean difference (SMD) with 95% confidence intervals (CIs) were used to weigh the ESs.^[19]^ The SMD values of 0.2, 0.5, and 0.8 were considered as small, medium, and large ESs of WBVT. *P* values less than .05 were considered to be statistically significant in our meta-analysis.

The Cochrane Q test for heterogeneity was performed and considered statistically significant if *P* ≤ 0.10. Heterogeneity was also quantified with the *I*^2^ statistic. Studies with an *I*^2^ statistic of 25% to 50% were recognized as having low heterogeneity.^[20]^ In cases of moderate or high heterogeneity, each study was rereviewed to identify whether any discrepancy could be identified, and subgroup analysis was performed. Priori specified sources of clinical heterogeneity were analyzed in the following subgroup analyses control intervention type (no treatment or sham vibration vs exercise interventions), frequency of WBV, magnitude of WBV, acceleration of WBV, cumulative dose of WBV, and measuring lean mass by dual-energy X-ray absorptiometry (DXA, whole body or lower limbs). Median values of continuous variables were used as cutoff values to group the trials with frequency, magnitude, acceleration, and cumulative volume.

## 3 Results

### 3.1 Characteristics of study

From 130 potentially relevant titles and abstracts identified, 10 RCTs were determined eligible.^[21–30]^ The majority of identified studies were excluded because they were not RCTs. The remaining RCTs were then primarily excluded because they did not obtain lean mass or muscle mass measurements. Figure 1 shows the search results and selection procedure. The RCTs included in our systematic review involved the following study population types: older people, younger adults, postmenopausal women, adolescents, and children. The details of each study are summarized in Tables 1 and 2.

**Table 1:**
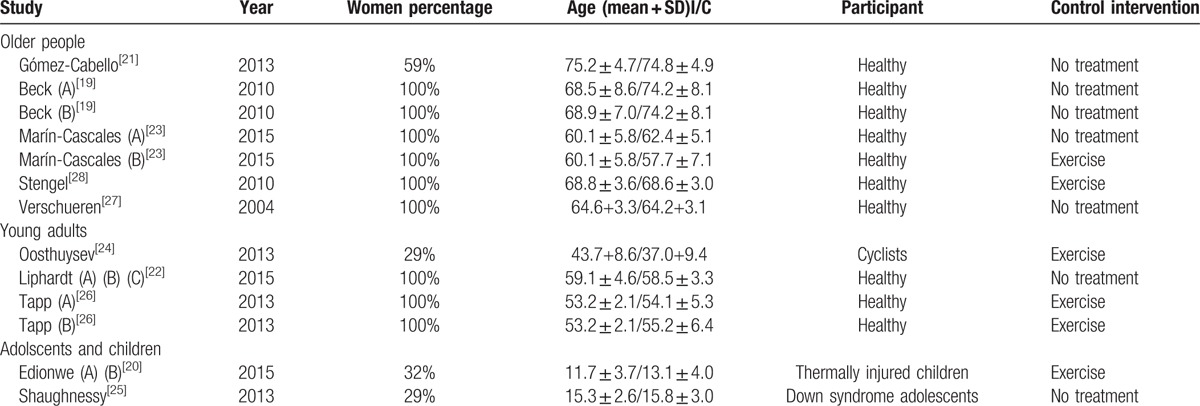
Description of included studies.

**Table 2:**
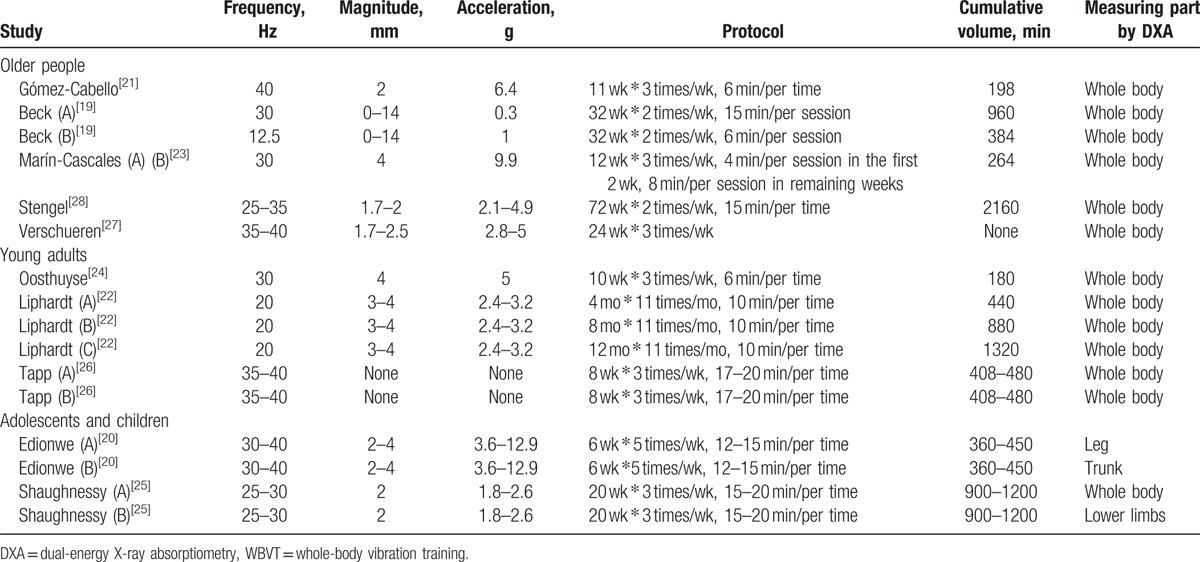
Characteristics of WBVT intervention and muscle mass or lean mass assessment.

### 3.2 Main analysis

#### 3.2.1 Older people

**Figure 3:**
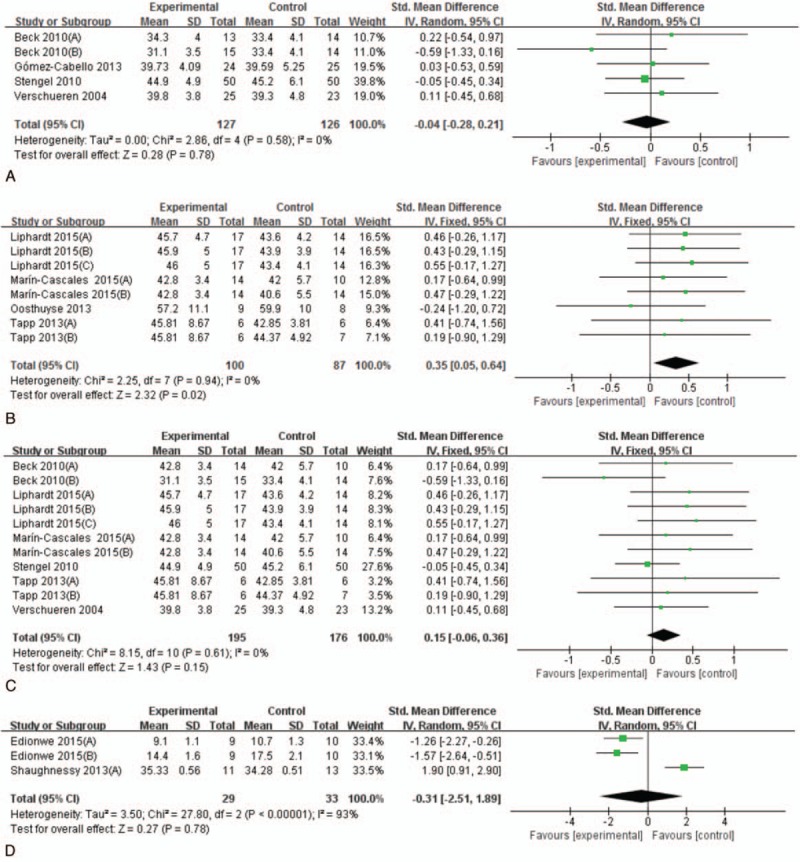
Primary analyses of whole-body vibration training (WBVT) effect on lean mass in different populations, (A) aged people, (B) young adults, (C) postmenopausal women, and (D) adolescents and children.

There were 4 trials in aged population.^[21,23,25,29,30]^ Study participants included 239 healthy nonathletic European population aged over 65 years old. The results from the SMD between experimental and control groups showed no significant differences (SMD = −0.04, 95%CI: −0.28, 0.21; Fig. 3A). Moreover, in all subgroup analyses of whole-body vibration effect on muscle mass or lean mass in older people, our results remained nonsignificant (see Table 3).

**Table 3:**
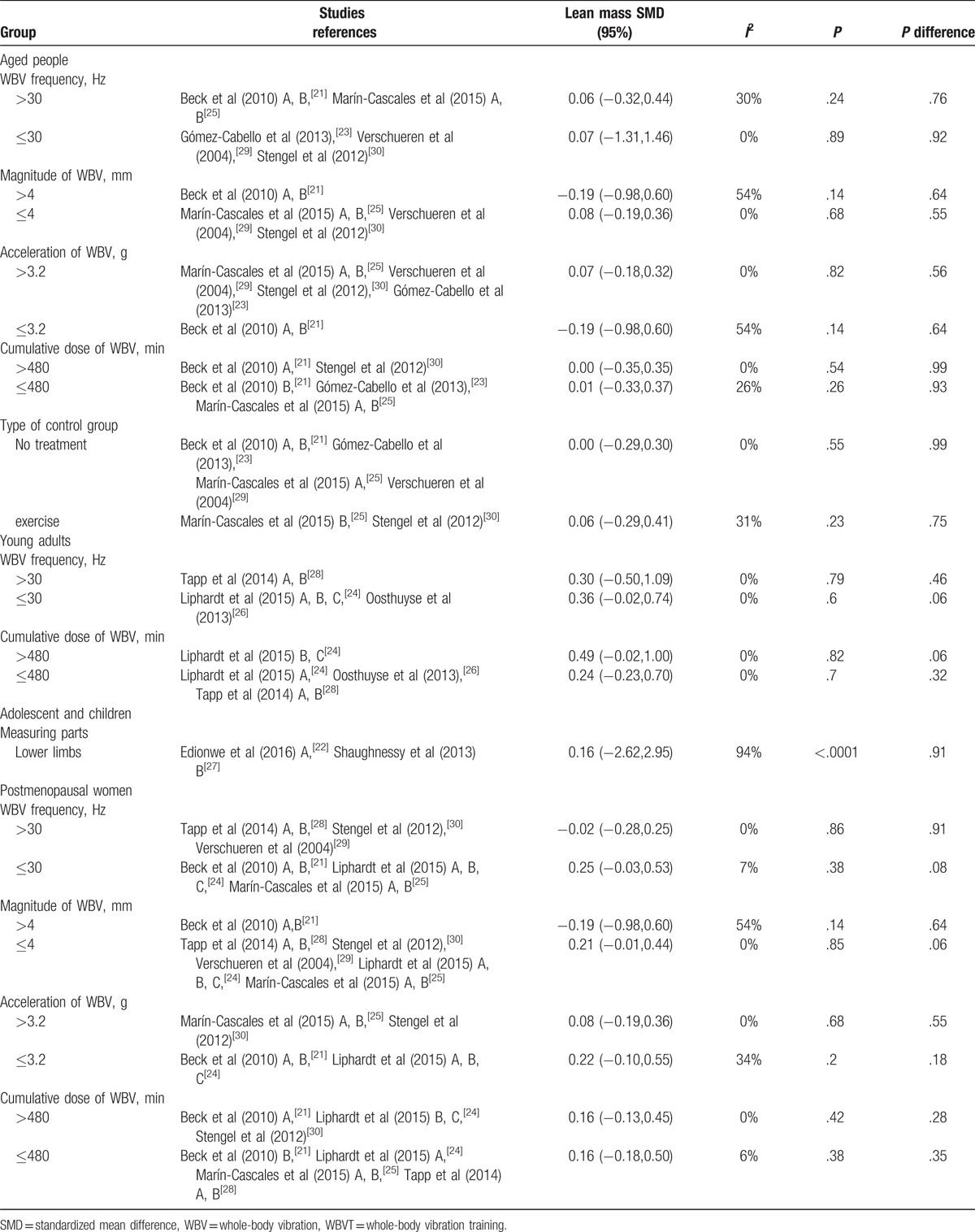
Subgroup analyses of WBVT effect on lean mass.

#### 3.2.2 Young adults

We extracted the effects of WBVT on muscle mass or lean mass in adults from 4 studies involving 103 adults aged under 65, which includes cyclists and nonathletic participants.^[24,26,28]^ Significant difference was found between experimental and control groups (SMD = 0.35, 95%CI: 0.05, 0.64; Fig. 3B). However, in all subgroup analyses of whole-body vibration effect on muscle mass or lean mass in young adults, our results turned nonsignificant (see Table 3).

#### 3.2.3 Postmenopausal women

There were 6 trials including 279 healthy nonathletic European women to evaluate the effects of WBV on muscle mass or lean mass.^[21,24,25,28–30]^ The results from the SMD between experimental and control groups showed no significant differences (SMD = 0.15, 95%CI: −0.06, 0.36; Fig. 3C). In all subgroup analyses of whole-body vibration effect on muscle mass or lean mass in older people, our results remained unchanged (see Table 3).

#### 3.2.4 Children and adolescents

There were only 2 trials including 43 children and adolescents to assess the influence of WBV on muscle mass or lean mass.^[22,27]^ Study participants included boys and girls aged 12 to 18 years with Down syndrome^[27]^ and thermally injured children.^[22]^ The results indicated that there were no significant differences of lean mass or muscle mass between experimental and control groups (SMD = −0.31, 95%CI: −2.51, 1.89; Fig. 3D). Moreover, when solely taking the muscle mass of lower limbs instead of the lean mass of whole body into account, the results stayed the same (SMD = 0.16, 95%CI −2.62, 2.95).

## 4 Discussion

The aim of this meta-analysis was to evaluate the existing literature regarding the effects of WBVT on lean mass or muscle mass in human. Our systematic evaluation of WBV found statistically significant improvement in lean mass or muscle mass in young adults (SMD = 0.35, 95%CI: 0.05, 0.64, *P* = .05). Although statistically significant, the effect size in adults was small (z = 2.32). No significant effects were found in other populations. Hence, it may be not proper to take WBVT for the purpose of maintaining muscle mass in the population of children, adolescents, older people, and postmenopausal women.

There were various protocols for testing WBVT which included different methods of application, vibration characteristics (eg, frequency, amplitude, and acceleration), training duration, and study population. After WBVT in older people and postmenopausal women, changes in muscle mass observed in some of the included studies seem to be independent of vibration characteristics. The use of different frequencies (>30 Hz or less) or different amplitudes (>4 mm or less) or different acceleration (>3.2 g or less) found similar effects on muscle mass. Studies with different cumulative volume of WBVT (>480 minutes or less) obtained similar results. Besides, when making a subgroup analysis grouping by control intervention type (no treatment or sham vibration vs exercise interventions) in older people, the results remained unchanged. In addition, we could discover that neither the lean mass in whole body nor in lower limbs measured by DXA have significant differences after WBVT in children and adolescents. In other words, there may be no different effects of WBV on different parts of body in children and adolescents. To sum up, we could draw our conclusion with more certainty that WBVT is not effective in improving muscle mass or lean mass in older people, postmenopausal women, adolescents, and children.

The older population may show greater increases in lower limb muscle activity during WBV exercise than their young counterparts, which means that they might benefit more from WBV exercises.^[31]^ Our analysis however shows that compared to the elderly, statistically significant improvement in lean mass or muscle mass was found in younger adults. Furthermore, excluding the athletic participants did not change the result, which may support the application of WBVT in young adults for the sake of maintaining or augmenting muscle mass. In accordance with older people and postmenopausal women, we could not find close relationships between the WBVT characteristics and the effects on muscle either. However, previous literatures show that different diameters of vibration may have diverse effects on muscle both in mice and human. High-frequency and high-acceleration WBVT may be more effective than low frequency in ameliorating aged-induced muscle loss in people and middle-aged mice.^[31,32]^ In various vibration frequencies, average electromyography root mean square activity of vastus lateralis was higher than in the no-vibration condition, and the electromyography root mean square was different in vastus lateralis muscle during WBVT conducted with diverse protocols.^[33]^ Therefore, further studies are required to explore the optimal protocol of WBV during clinical practice.

Although not fully understood, it has been proposed that neural and endocrine reactions may both mediate the training effect. Various neural mechanisms have been implicated in WBV-induced increased muscle activity. The most frequently cited mechanism underpinning the WBV response is a reflex muscular contraction termed the tonic vibration reflex, which refers to a sequence of rapid muscle stretching that occurs when applying vibration and that triggers muscle spindles.^[34,35]^ Other mechanisms of improved muscle function following vibration include enhanced corticospinal excitability and intracortical processes.^[36]^ Furthermore, it has been found that exposure to vibration produces a sharp increase in blood levels of hormones such as testosterone and growth hormone.^[37]^ These endocrine changes would partly explain the alteration in lean mass after vibration training.

There were significantly different effects of WBVT among different age groups in this meta-analysis, which suggests that different stage of age may be a vital factor in observed changes in muscle mass after WBVT. Considering the precise mechanisms that may underpin the relationship between age and the effects of WBVT are yet to be fully elucidated, another important future research is expected to be conducted.

### 4.1 Limitation

There are several limitations to our study: the small number of included studies due to the few publications in the existing literature that focused on the effect of WBVT intervention on lean mass, especially the children and adolescents (2 RCTs, n = 43) populations; the number and percentage of males are too low in the population of older people and young adult in the study we included; the included studies used DXA scans to obtain lean mass which do not purely measure skeletal muscle mass; and the training attendance was not completely accordant with the designed plans.

## 5 Conclusion

This is the first meta-analysis of the effects of WBV on lean mass or muscle mass in humans with no restrictions on age and sex. Our evaluation demonstrated that WBVT could lead to improvement in lean mass or muscle mass in younger adults but not in other populations, which seemed not to depend on the vibratory parameters (frequency, amplitude, and acceleration), dose and control intervention type (no treatment or sham vibration vs exercise interventions). Rigorous and well-planned trials with larger samples should be conducted to determine the effectiveness of WBV in human and to explore the valid and optimal project in respective population.
